# Fire Resistance of Foamed Concrete for Discontinuous Partition Filling

**DOI:** 10.3390/ma17061315

**Published:** 2024-03-13

**Authors:** Paweł Sulik, Bożena Kukfisz, Adriana Dowbysz, Agata Oszczak-Nowińska

**Affiliations:** 1 Fire Research Department, Building Research Institute, 00-611 Warsaw, Poland; p.sulik@itb.pl; 2 Institute of Chemistry, Military University of Technology, Kaliskiego 2 Street, 00-908 Warsaw, Poland; agata.oszczak@wat.edu.pl; 3 Department of Chemistry, Biology and Biotechnology, Bialystok University of Technology, 15-351 Bialystok, Poland; adriana.dowbysz@pb.edu.pl

**Keywords:** foam concrete, density, insulating fire temperatures, fire resistance

## Abstract

Lightweight concrete exhibits many advantages over traditional concrete such as lower density and thermal conductivity and an easier, cheaper, less energy-consuming manufacturing process. In order to extend its applications, there is a need to study its behavior in fire situations. Due to that, the aim of this study was to assess the fire resistance of foam concrete, depending on its thickness and the foaming process applied. Fire resistance was assessed according to EN 1363-1. The results indicate the usefulness of foam concrete in terms of isolating fire temperatures for discontinuous partition filling that are consequently a real alternative to dedicated solutions in the field of passive fire protection. The density of foam concrete was shown to have a large effect on the ability to insulate fire temperatures with a standard material preparation process. It was also noted that changing the method to continuous foam feeding may result in the achievement of similar values while maintaining foam concrete low density.

## 1. Introduction

The increasing number of fires with a range of consequences such as death, loss of property, and financial issues has meant that fire resistance research has continually attracted the attention of scientists. As fire is the cause of the reduction in material properties and load-bearing capacity of structures [1], construction material regulations include fire resistance tests which provide an assessment of load-bearing characteristics and fire separation properties. The tests are performed in large furnaces with reference to a standard temperature–time curve [2] defined by the equation:

$$\theta_{\infty }=20+345{log}_{10}\left(8t+1\right),$$

where t—time [min], and $\theta_{\infty }$—the gas temperature in the fire compartment (°C) [3].

Many researchers have also studied the material properties of concrete subjected to elevated temperatures. According to Arioz [4], concrete made of laterite and quarry dust exposed to temperatures of 250–1200 °C gradually loses its weight, which sharply reduces to over 800 °C. The relative strength of the concrete also reduces with the increase in temperature.

Nanotechnology has also gained more interest in terms of nanomaterial applications in indirect fire protection [5]. The research conducted by Kanagaraj et al. [6] on the influence of nanomaterials such as nano-cement, nano-silica-fume, nano-fly-ash, and nano-metakaolin on the impact strength of concrete, revealed that unheated materials exhibited an increased impact strength compared to concrete without additives. However, samples subjected to elevated temperatures in a range of 250–1000 °C exhibited a decrease in impact strength, with the worst performance obtained for concrete containing metakaolin. It was found that the optimal amount of added nanomaterials should not exceed 10 wt.%.

Studies of material behavior, such as steel or concrete, under fire conditions, can be carried out both experimentally and analytically, including a number of studies such as those conducted by Oliveira et al. [3] on hot-rolled steel profiles, Li et al. [7] on steel fiber reinforced concrete beams, Issa et al. [8] on reinforced concrete beams, or Schabowicz et al. [9] on façade materials, indicating that the results of tests performed by both methods are in agreement.

In order to identify the relationships between the keywords (concrete and fire), a co-occurrence analysis was conducted based on articles ranked in the Web of Science Core Collection database published from 2014 to 2024 (1138 elements). A model and visualization of the bibliographic network were prepared with the use of VOSviewer version 1.6.20.

A network visualizes 44 keywords grouped in three clusters. Table 1 presents 10 of the most frequently occurring keywords along with their number of links, total link strength, and occurrences.

**Figure 1:**
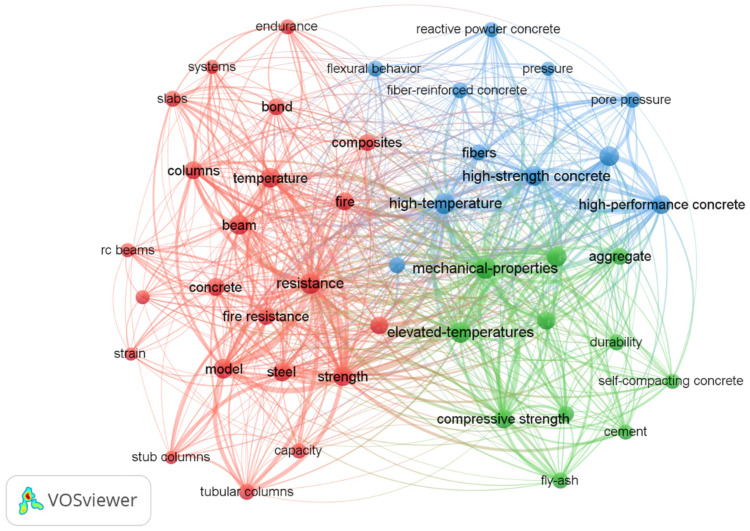
Analysis of keyword (concrete and fire) co-occurrence from 2014 to 2024 (keyword co-occurrence threshold of 15).

Based on keyword co-occurrence analysis, it can be observed that elevated-temperature research of concrete containing elements, such as beams or columns, and other types of concrete, such as high-performance concrete, is gaining much attention. The fire behavior and fire resistance of concrete and its microstructure are also a focus of attention. However, as can be seen from the network (Figure 1), foam concrete has not been yet included in the keyword co-occurrence in this study.

Due to shrinkage, typical concrete is not an optimum material for filling construction discontinuities, especially those with complex, irregular shapes. A much better material is rock mineral wool or system solutions for penetration seals. Plasterboard is a very good solution in terms of fire resistance, although for larger surfaces such as ceilings and walls. Foamed concrete is therefore closest to concrete in terms of fire resistance: due to its porous structure and properties, it fills any discontinuities more easily and is not subject to spalling.

Classified as lightweight, foam concrete is a cellular type of concrete (LCC) with a density range of 300–1600 kg/m^3^. The role of density is important in terms of strength and thermal conductivity, which was studied by Compaoré et al. [10]. Although the decrease in density results in a decrease in strength, the research indicates the reduction in this phenomenon due to enhancement in the growth of amorphous and recrystallized phases inside the air voids. The air-void structure of foam concrete is obtained by introducing foam, prepared from foaming agents, water, and air, into cement mortar. Foam concrete is valued for its mechanical properties, low cement content, low aggregate consumption, and significant thermal insulation performance. Moreover, it is considered a cost-effective material for lightweight structural components’ production such as that of structural elements, partitions, fills, and road embankments [11,12], due to the weight reduction of up to 80% compared to conventional concrete. A comparison of different types of concrete is presented in Table 2.

**Figure 2:**
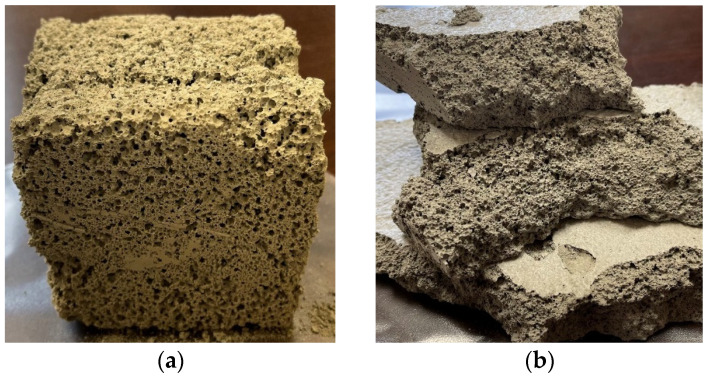
Examples of foam concrete structures: (**a**) stable, acceptable; (**b**) brittle, non-acceptable.

The density of foam concrete, its stability, porosity, and fluidity depend on the content and structure of air voids (cells). There are a variety of foaming agents, including glue resins, detergents, resin soap, and saponins. However, the most frequently used are synthetic and protein-based ones. The latter enables the formation of a stronger, more stable, and more compact air-void network, while the former is characterized by greater expansion. In addition, they are cheaper, easier to use than protein-based foaming agents, and require a lower amount of energy for safe storage. Examples of foam concrete structures are depicted in Figure 2. These images illustrate various samples of foamed concrete with different compositions and densities, representing the preliminary phase of experimentation conducted before selecting elements for the studies outlined in the article. In Figure 2a, a sample with an acceptable structure is shown which remained intact upon handling after setting. Conversely, in Figure 2b, a sample with inadequate mechanical properties is displayed which disintegrated upon handling.

Foam concrete is also distinguished by its great acoustic and thermal insulation properties, fire resistance, and water resistance, compared to conventional concrete [29]. Another advantage is that it may be used as a filler due to its strong energy-absorption properties [30].

Although the physical and mechanical parameters are well recognized and described in the literature [13,31,32], in terms of fire characteristics there is a clear deficit of information useful in the design process of this type of construction. Despite belonging to the group of concretes, unlike plain concrete, whose fire parameters and behavior are well recognized [20], the presence of air-void cells in foam concrete should positively affect its fire behavior, if only by reducing and perhaps eliminating explosive spalling.

Concrete itself is non-flammable and does not spread fire [33]. Difficulties arise in the evaluation of the fire resistance of foamed concrete.

Rybakov et al. [34] studied the effects of high temperature on monolithic foam concrete with a density of 200 kg/m^3^ produced from Portland cement 42.5 N, foaming agent, and water used in lightweight steel concrete structures. A combustibility test was performed on samples with a diameter of 43 mm and a height of 50 mm. Although the results showed that this particular foam concrete is a non-combustible material, it did not contain any additives, and the test specimens were small.

Yew et al. [35] investigated the effects of the addition of oil palm shells (OPS) on the strength and fire resistance properties of foam concrete produced from Ordinary Portland Cement (type I), foaming agent, and water. A fire resistance test with the use of a Bunsen burner was performed on samples of size 300 mm × 300 mm × 50 mm. Decreasing the density of concrete as the OPS content increased had the effect of reducing the compressive strength of materials. However, the OPS addition resulted in a slower increase in the material temperature from 80 °C to 87 °C, due to their greater porosity and slower heat exchange.

Studies by Awoyera et al. [36] indicated that the incorporation of ceramics and mineral additives may positively affect the fire resistance of foam concrete produced from Portland cement 42.5, sand, ceramic waste, a foaming agent in the form of an aluminum powder, plasticizer, and water. Samples of 150 mm cubes were exposed for an hour to temperatures of 400 °C, 500 °C, and 600 °C in a furnace. On the one hand, the results showed that between 500 °C and 600 °C, the contraction and disintegration of particles caused a significant decrease in the residual compressive strength of foam concrete. On the other hand, the increasing content of ceramics improved the fire resistance properties of foam concrete.

An interesting study has been conducted by Othuman [37] on the mechanical properties of foam concrete at temperatures of 20–800 °C. The results indicated that stiffness loss mainly occurs after achieving a temperature of 90 °C, regardless of the density of foam concrete, which varied from 500 to 1500 kg/m^3^, which gives rise to the assumption that the loss of stiffness is directly affected by internal cracking.

As can be seen from the literature review, there are only a few articles on this subject concerning small samples, which do not allow a reliable assessment in terms of fire resistance [38]. Due to the lack of research concerning the fire resistance of foam concrete studied at defined heating and pressure conditions, the aim of this study was to assess and compare the different behavior of several foam concrete materials in the same fire conditions, as well as to make a comparison of the effect of the density of foam concrete on its ability to insulate fire temperatures. The solutions evaluated, in terms of fire safety, are assessed in terms of reaction to fire and fire resistance. In terms of fire safety and reaction to fire classes, because of decisions made by the European Commission regarding materials like concrete, no verification was needed. Therefore, an analysis wasn’t included in the article. In the case of fire resistance, experimental verification was carried out according to the guidelines of EN-1363-1:2020 Fire resistance tests—Part 1: General requirements [39], the basic standard for this type of testing.

## 2. Materials and Methods

### 2.1. Materials

The foam concrete specimen molds used were made of fireproof silicate-cement panels with a wide range of applications in fire protection, including the construction of self-supporting smoke and ventilation ducts, cable route enclosures, and the construction of partitions that are fire separations (Promatect L-500 (Promat, Stelmachowo, Poland), thickness of 25 mm, external dimensions of 400 mm × 400 mm × 200 mm). The mass of the bottom of the mold lay in a range of 2.04 kg to 3 kg, and it stood as protection during transport. The calculated mold frame weight was 3.75 kg, and the actual mold frame weight lay in the range of 3.85 kg to 4.29 kg.

**Figure 3:**
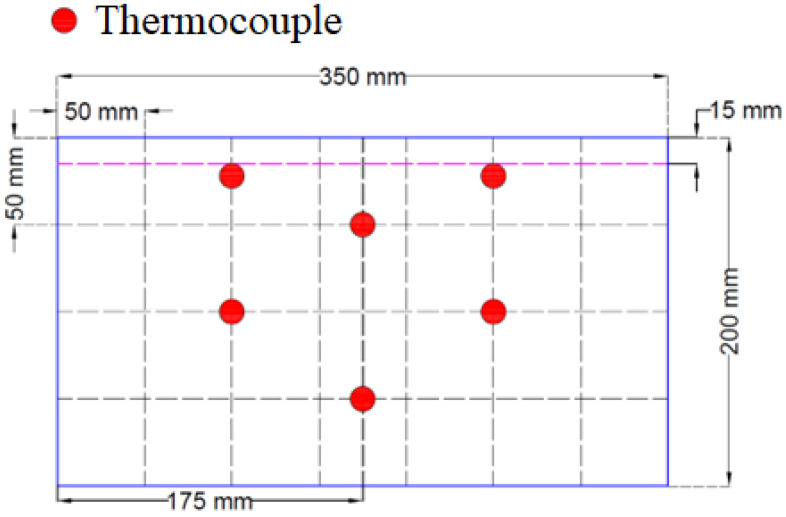
Distribution of thermocouples inside the sample.

**Figure 4:**
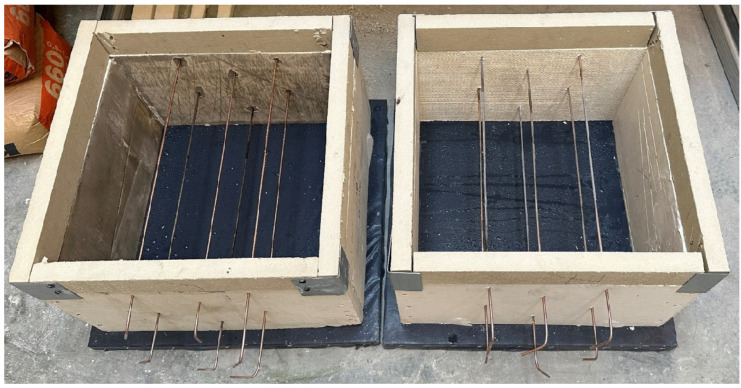
Molds with elements essential to the insertion of thermocouples.

During mold preparation, six elements were placed in order to allow the insertion of K-type thermocouples (Jumo, Wrocław, Poland). The distribution of elements and molds are presented in Figure 3 and Figure 4, respectively.

The foam was produced using a generator constructed according to the concept of all the authors, consisting of a foaming agent pump, aeration system, foam generator, and foaming agent tank. The foam was produced from Pianotwór (MEEX-AG, Chrzanów, Poland), a lightweight concrete foaming agent used in the mechanical manufacture of foam, causing the closure of air bubbles in the resulting mixture. This contributes to frost resistance and tightness, does not degrade the degree of flammability, reduces the weight of cement mortars, and provides high parameters for thermal and acoustic insulation. Foam was produced from a 3% solution of foaming agent with water, at the air pressure of 0.4 MPa.

Samples were prepared under laboratory conditions using a quantitative sampling method in various combinations to achieve the assumed parameters related to the density of the finished product. All ingredients were mechanically mixed after weighing. Samples made of Material 5 were made in a continuous operation system using a concrete pump and a foam generator connected by a stirrer. These samples were made on the premises of the concrete plant. All samples were prepared according to EN 1363-1 Fire resistance tests—Part 1: General requirements [39].

**Figure 5:**
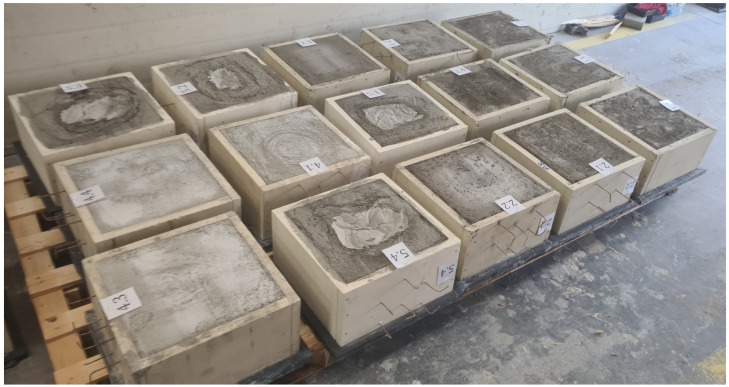
Distribution of thermocouples inside the samples.

Three samples of each material were prepared. Samples in molds are presented in Figure 5. The detailed composition of the foam concrete materials is presented in Table 3.

The weight of all samples was determined with the usage of a weighing machine (C315.P.60, RADWAG, Radom, Poland). The density was calculated based on the internal mold volume. Masses and densities of all samples are presented in Table 4.

Samples were conditioned for six weeks at a temperature range of 14.8 °C to 21.5 °C and a humidity range of 26% to 71%.

### 2.2. Methods

The fire resistance tests were performed according to EN 1363-1 [39], with the usage of a Spark furnace (Seco/Warwick, Świebodzin, Poland, with dimensions of 3.7 × 3.7 m, maximum power of 2.7 MW, and equipped with a control system (Pumpa, Łuczyce, Polska)).

**Figure 6:**
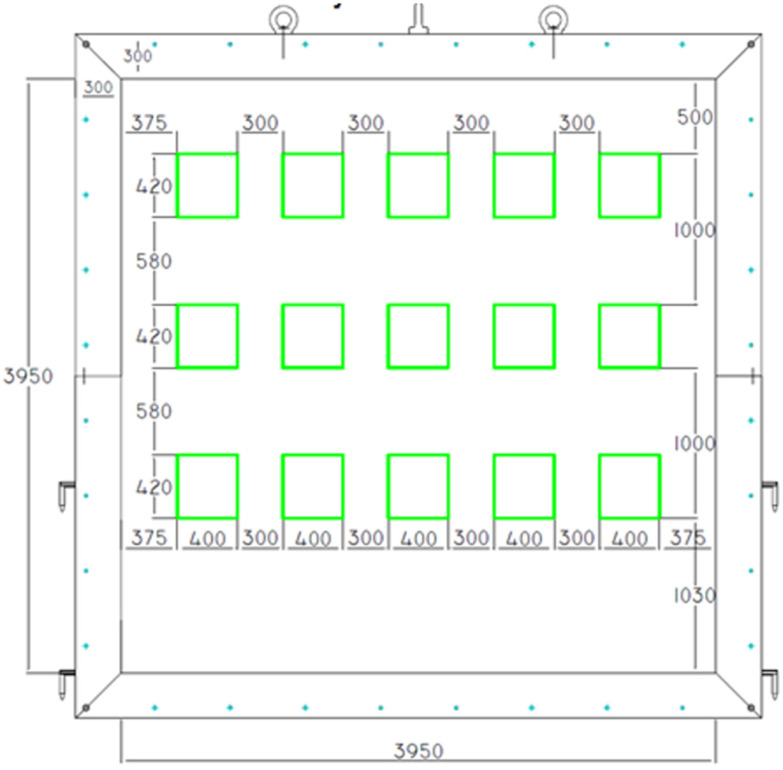
Distribution of samples in the test wall.

**Figure 7:**
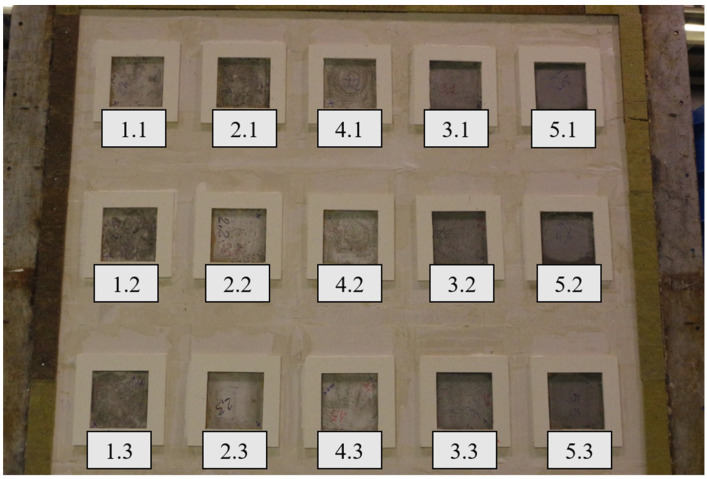
Samples were placed in the support structure (heated side).

Samples were built into a support structure made from autoclaved cellular concrete with a density of 600 kg/m^3^, using adhesive cement-base mortar. The thickness of the fastening structure was 240 mm. The joints at the border between the autoclaved cellular concrete and the steel frame were sealed with mineral wool. Due to the different pressure conditions in the furnace, samples of one type were arranged vertically, assuming a pressure of 20 Pa prevailing at the top edge of the highest row of samples. The distribution and arrangement of samples in the test wall are presented in Figure 6 and Figure 7.

**Figure 8:**
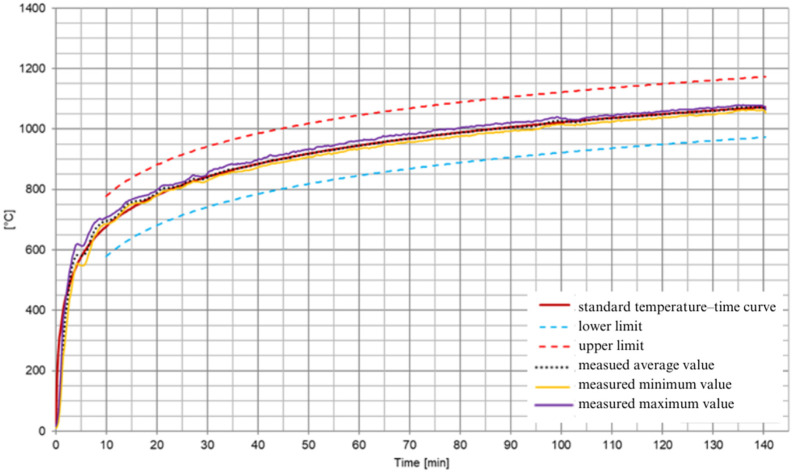
Temperatures of the furnace heating conditions.

The temperature within the furnace was controlled according to the standard temperature–time heating curve [40] (Figure 8).

## 3. Results

**Figure 9:**
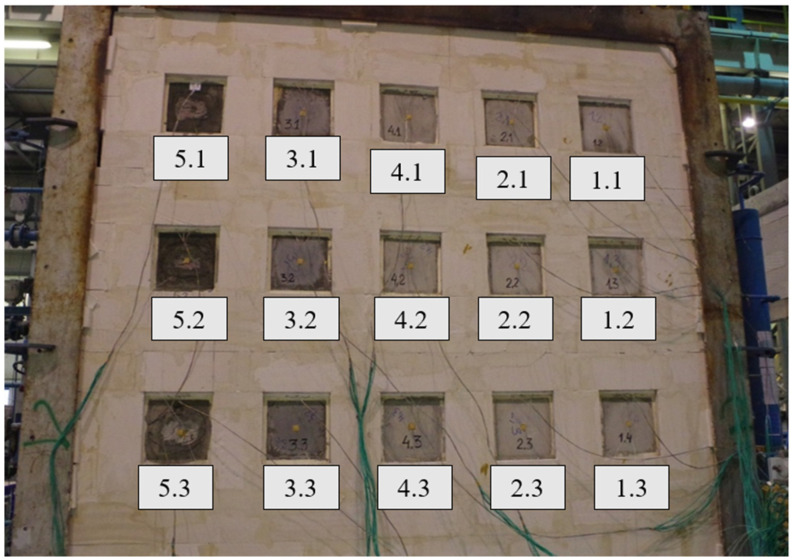
Samples at 60 min of the measurement (non-heated side).

**Figure 10:**
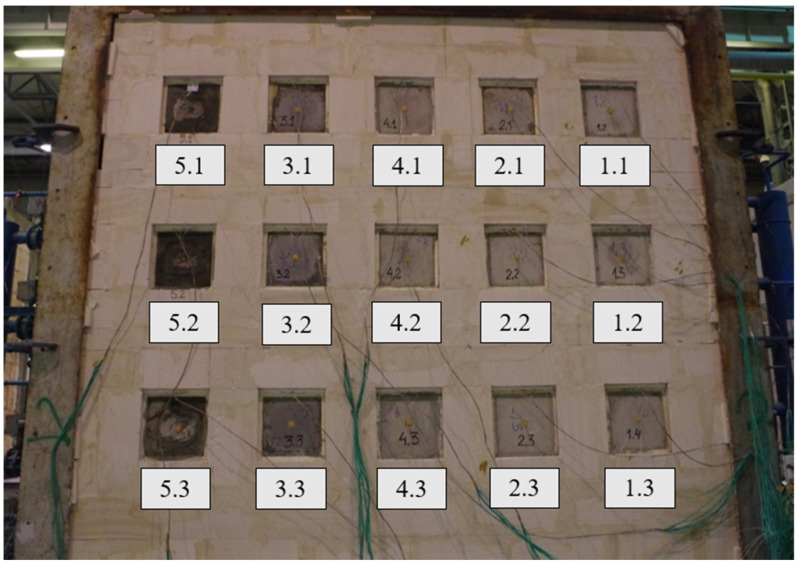
Samples at 120 min of the measurement (non-heated side).

**Figure 11:**
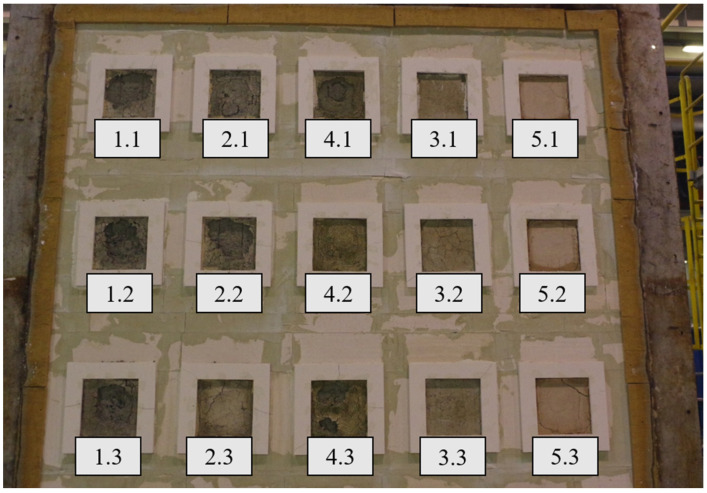
Samples after the measurement (heated side).

**Figure 12:**
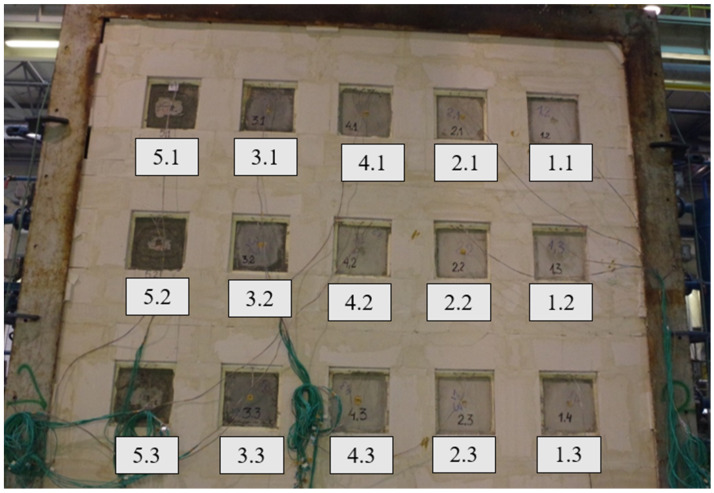
Samples after the measurement (non-heated side).

Figure 9, Figure 10, Figure 11 and Figure 12 present the samples placed in the support structure (non-heated side) at 60 and 120 min of the measurement, as well as the samples after the test (140 min) from the heated and non-heated sides. 

**Figure 13:**
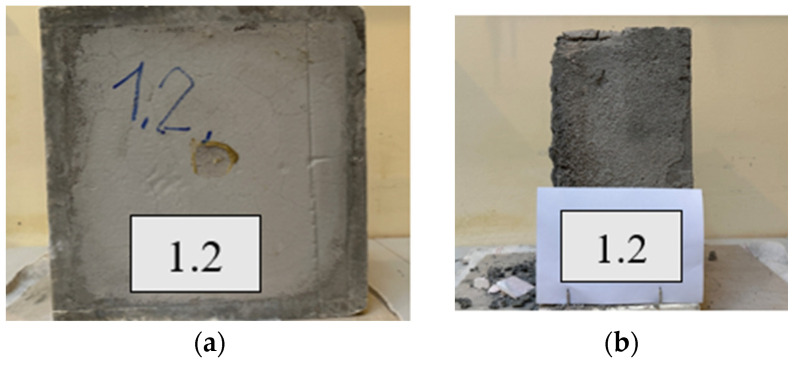
Sample 2 of Material 1 after testing: (**a**) from the heated side, (**b**) in cross-section.

The most significant degradation was observed in the sections of the samples facing the heat source (Figure 13a), up to 5 cm into the sample (Figure 13b), while the remainder showed no visible signs of structural changes or cracking. The foamed concrete behaved similarly to autoclaved aerated concrete, where only the outer layer facing the fire exhibited degradation. Due to the mineral nature of the sample, no combustion occurred within it (the material belongs to fire reaction class A1 and is treated as non-combustible), with primarily chemical transformations occurring only after exceeding the critical temperature for concrete.

The graphs of the time course of temperature for Materials 1–5 are presented in Figure 14, Figure 15, Figure 16, Figure 17, Figure 18, Figure 19, Figure 20, Figure 21, Figure 22, Figure 23, Figure 24, Figure 25, Figure 26, Figure 27 and Figure 28. The N (ISO-834) curve stands for the standard temperature–time curve [41]. A maximum temperature rise limit of 180 K above the initial average temperature was taken as a criterion for the effectiveness of fire temperature isolation, according to EN 13501-2:2023 [42].

For Material 1, with a density of 624.08 ± 11.31 kg/m^3^, confirmation of the effectiveness of insulating fire temperatures was obtained in the range of 47 to 87 min for a thickness of 50 mm. For thicknesses of 100, 150, and 200 mm, the effectiveness was confirmed for the entire duration of the test (140 min).

**Figure 17:**
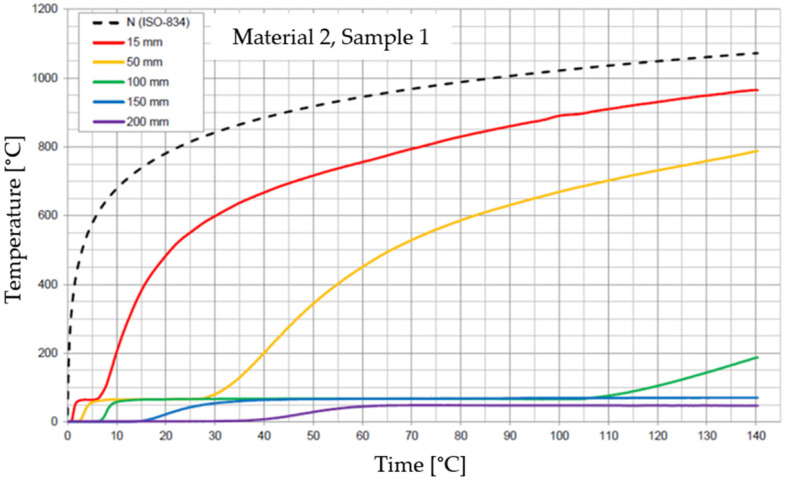
Temperature time courses for Sample 2.1.

**Figure 18:**
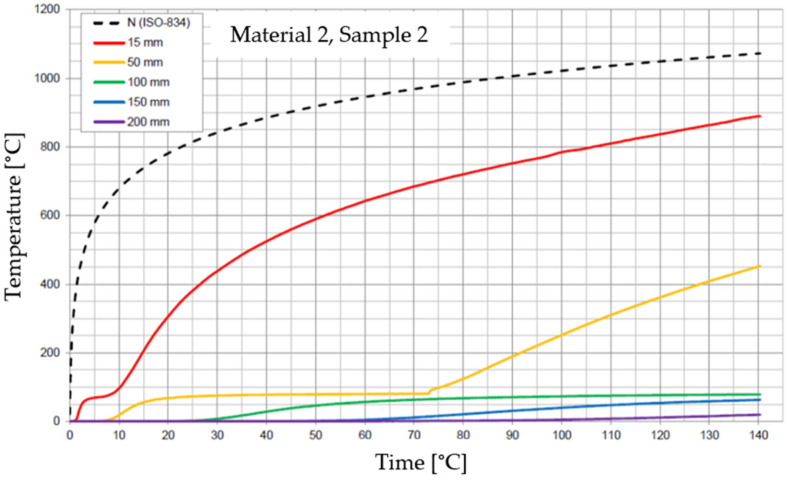
Temperature time courses for Sample 2.2.

**Figure 19:**
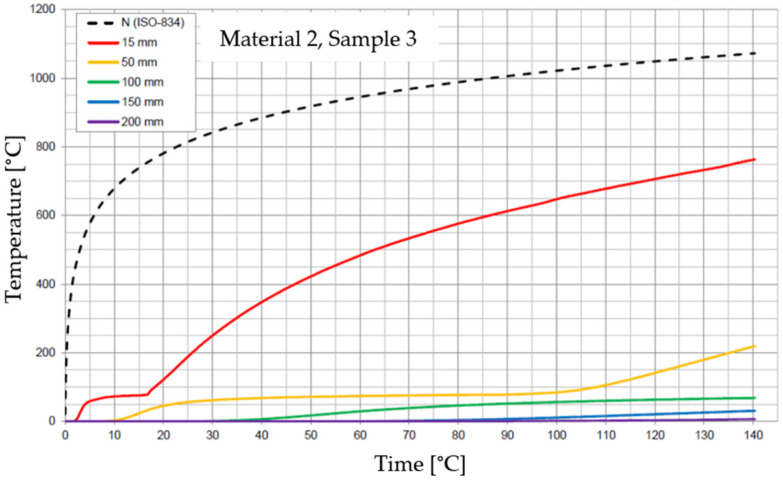
Temperature time courses for Sample 2.3.

For Material 2, with a density of 659.86 ± 8.57 kg/m^3^, confirmation of the effectiveness of insulating fire temperatures was obtained in the range of 38 to 130 min for a thickness of 50 mm. For a thickness of 100 mm, effectiveness was confirmed from 138 min to the entire test duration. For a greater thickness of material (150 and 200 mm), the effectiveness was confirmed for the entire duration of the test.

**Figure 20:**
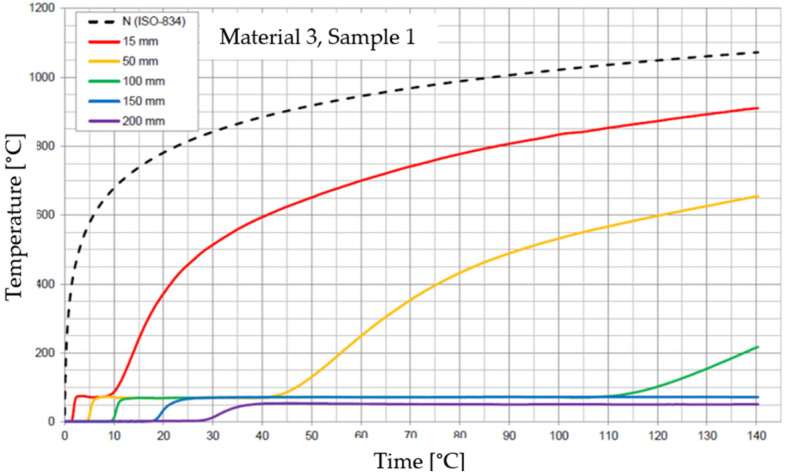
Temperature time courses for Sample 3.1.

**Figure 21:**
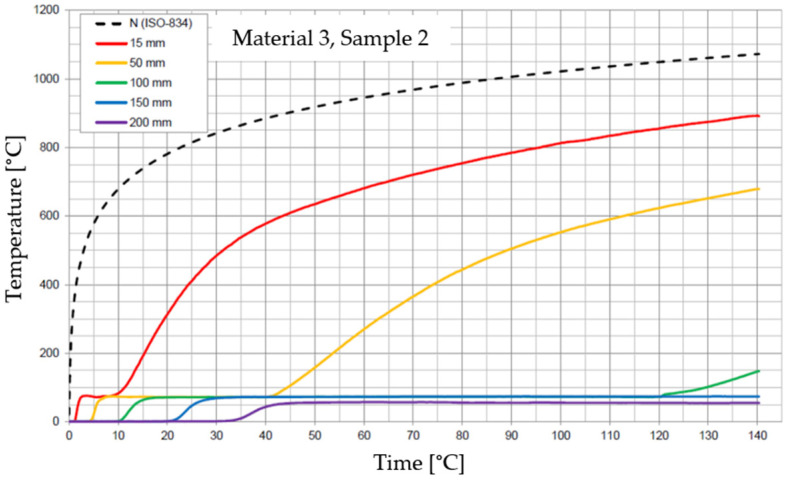
Temperature time courses for Sample 3.2.

**Figure 22:**
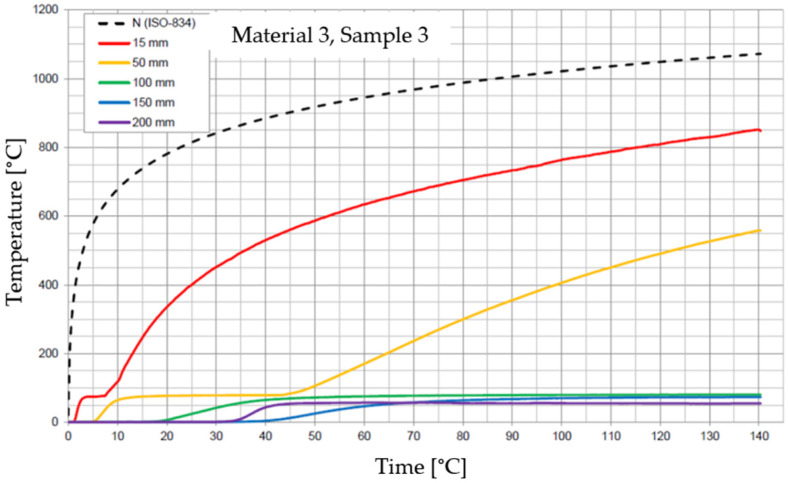
Temperature time courses for Sample 3.3.

For Material 3, with a density of 490.75 ± 22.69 kg/m^3^, confirmation of the effectiveness of insulating fire temperatures was obtained in the range of 53 to 63 min for a thickness of 50 mm. For a thickness of 100 mm, effectiveness was confirmed from 135 min to the entire test duration. For a greater thickness of material (150 and 200 mm), the effectiveness was confirmed for the entire duration of the test.

**Figure 23:**
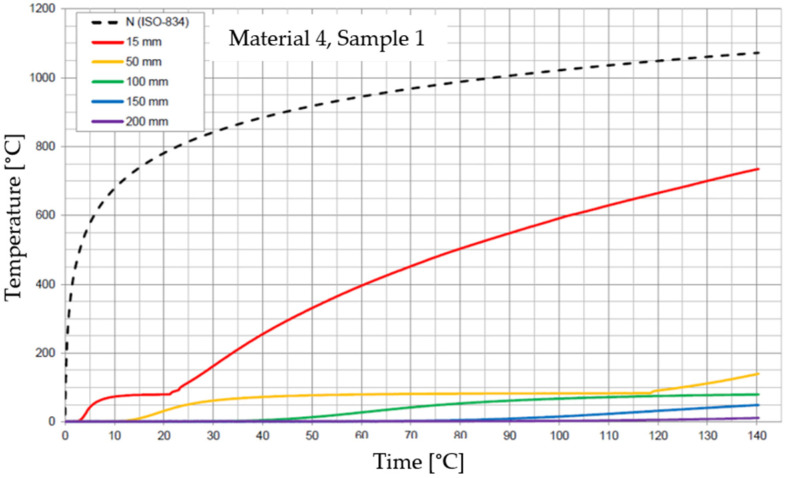
Temperature time courses for Sample 4.1.

**Figure 24:**
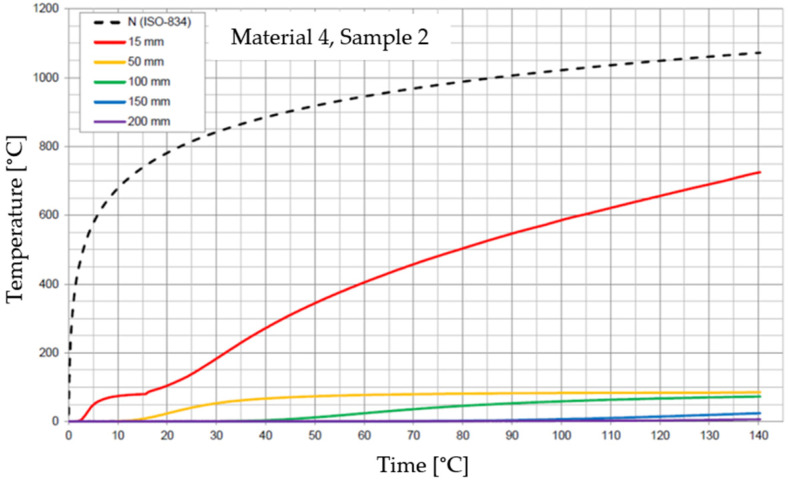
Temperature time courses for Sample 4.2.

**Figure 25:**
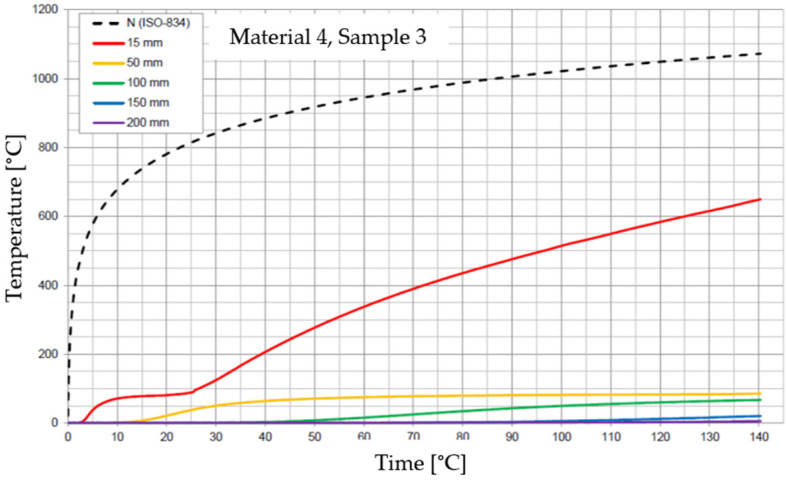
Temperature time courses for Sample 4.3.

For Material 4, with a density of 826.94 ± 31.37 kg/m^3^, confirmation of the effectiveness of insulating fire temperatures was confirmed for the entire duration of the test for all thicknesses of material, excluding the thickness of 15 mm.

**Figure 26:**
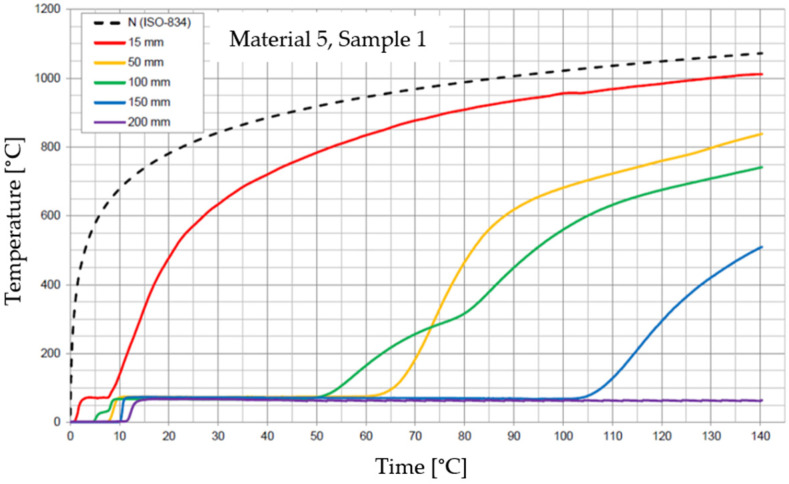
Temperature time courses for Sample 5.1.

**Figure 27:**
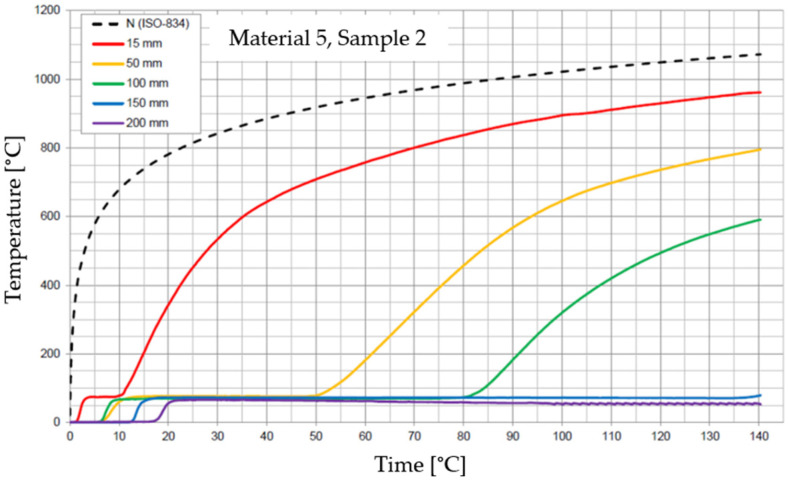
Temperature time courses for Sample 5.2.

**Figure 28:**
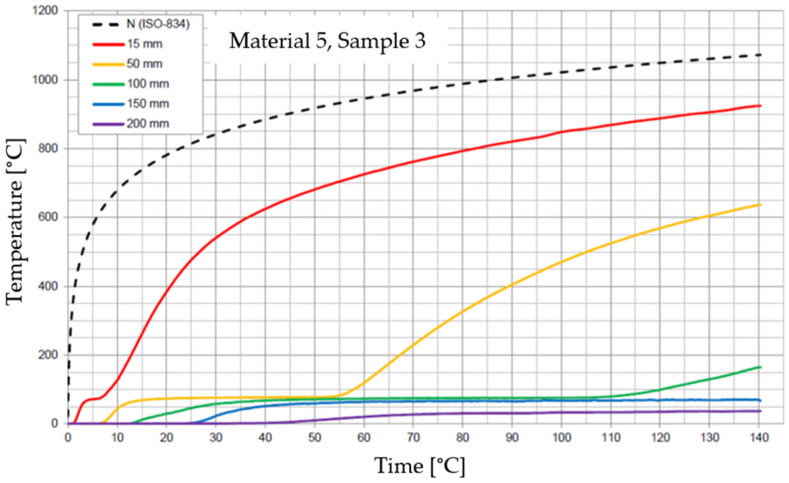
Temperature time courses for Sample 5.3.

For Material 5, with a density of 302.04 ± 8.53 kg/m^3^, confirmation of the effectiveness of insulating fire temperatures was obtained in the range of 60 to 70 min for a thickness of 50 mm. For a thickness of 100 mm, effectiveness was confirmed from 62 min to the entire test duration. For a greater thickness of material (150 and 200 mm), the effectiveness was confirmed for the entire duration of the test.

Interestingly, small discrepancies in the results for samples of this material were recorded at the 15, 50, 150, and 200 mm thicknesses, despite large discrepancies in the results recorded for Material 5 at the 100 mm thickness.

**Figure 29:**
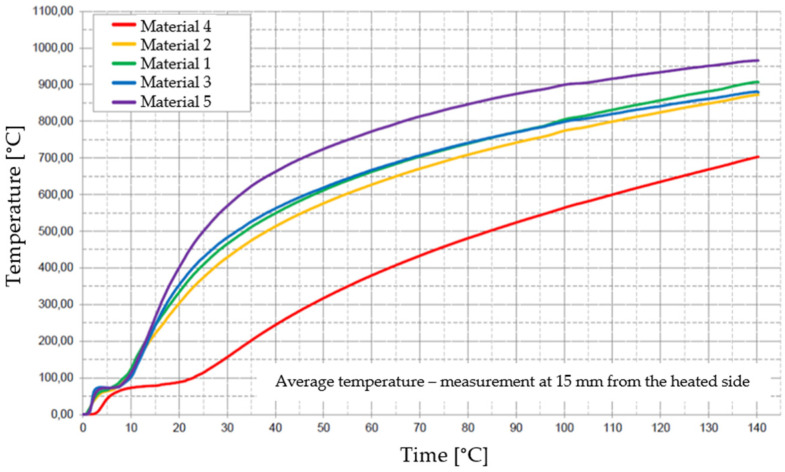
Average temperature time courses for materials studied at a thickness of 15 mm.

**Figure 30:**
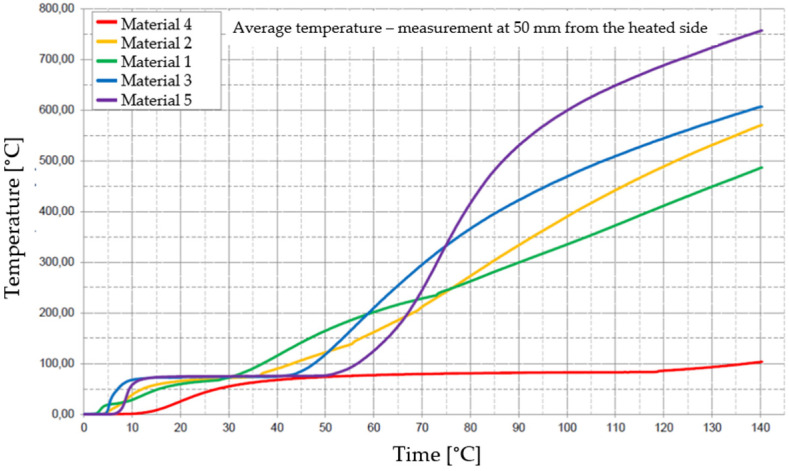
Average temperature time courses for materials studied at a thickness of 50 mm.

**Figure 31:**
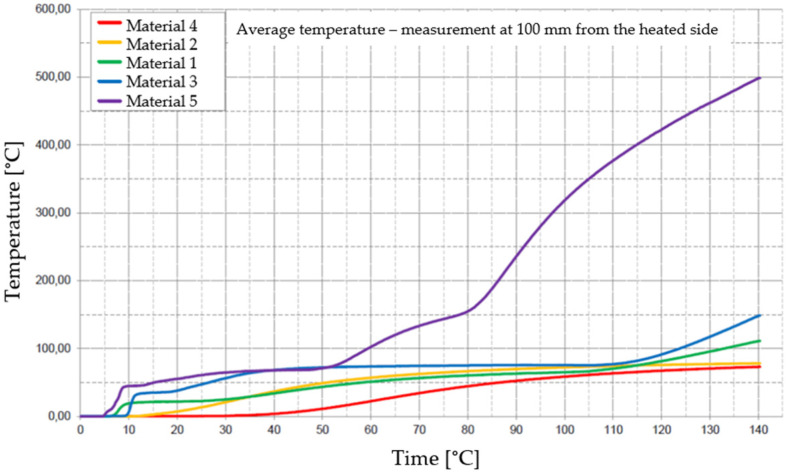
Average temperature time courses for materials studied at a thickness of 100 mm.

**Figure 32:**
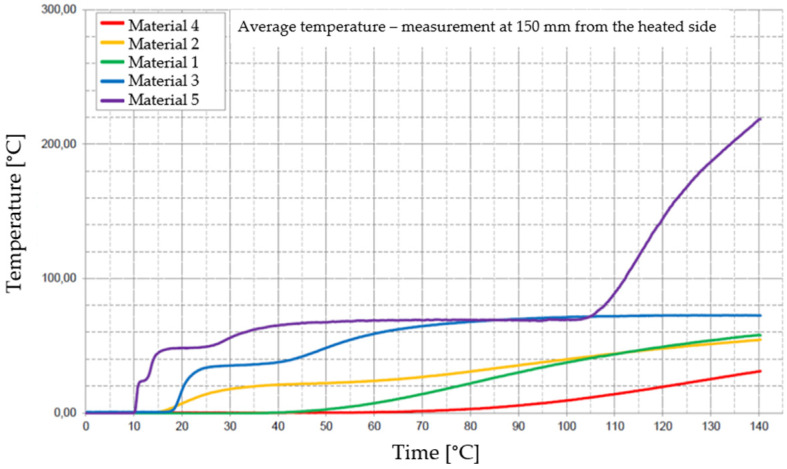
Average temperature time courses for materials studied at a thickness of 150 mm.

**Figure 33:**
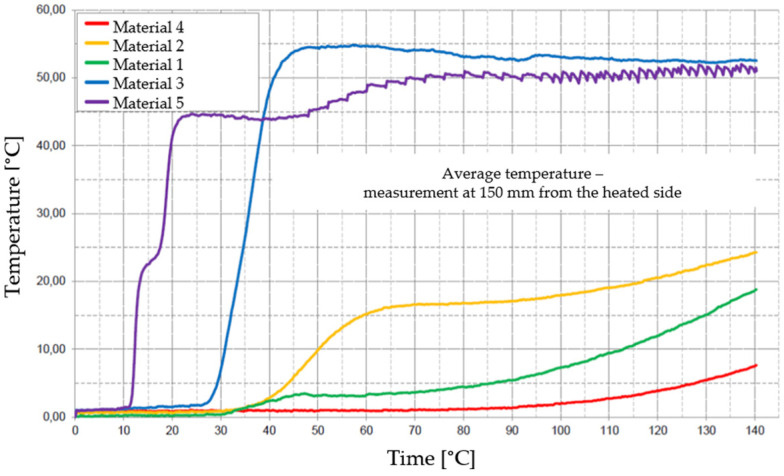
Average temperature time courses for materials studied at a thickness of 200 mm.

Figure 29, Figure 30, Figure 31, Figure 32 and Figure 33 represent the average time courses of temperature for the five materials studied at different thicknesses.

All findings from the experimental studies are summarized in Table 5. 

The most reproducible results were obtained for Materials 3 and 4, which were composed of cement 32.5 R and 42.5 R, respectively. As can be seen, the increase in the foam amount contributes to the reduction in their recurrence (Materials 2 and 1), especially at a thickness of 50 mm.

The results obtained in terms of insulating fire temperatures (fire insulation), as described in the article, are very satisfactory. Research in this area is continuing, with the inclusion of a larger specimen size and a narrowing of the density range to between 800 and 1400 kg/m^3^, due to the brittleness of lower-density specimens.

At this stage of the research, the mechanical resistance of the hardened foam concrete proved to be the biggest problem. It was noted that higher-density specimens behaved much better in this respect and, at the same time, better insulated against fire temperatures, which set the stage for further research. In the case of low-density foam concrete, it is advisable to use such solutions in areas not exposed to mechanical damage where, at the same time, mass plays a key role such as in improving the fire resistance of box ceilings by filling voids.

As mentioned above, the results obtained are very satisfactory. Foam concrete will not replace system solutions for penetration seals or sealing linear joints, although it has the potential to fill the gap where system solutions are not applicable. It is estimated that foam concrete can be successfully used to fill construction discontinuities in concrete, reinforced concrete, and masonry structures, primarily in ceilings and walls, with a particular focus on existing, converted buildings.

## 4. Conclusions

The test results obtained show great potential in insulating the fire temperatures of foam concrete, despite the high heterogeneity of samples. Thus, the main points from the above discussion are as follows.

- For all materials, the effectiveness of insulating fire temperatures for the entire duration test was confirmed for material thicknesses of 150 and 200 mm.
- Considering materials with a thickness of 100 mm, confirmation of effectiveness throughout the test was obtained for Materials 1 and 4. At the same thickness, Materials 2 and 3 maintained their effectiveness up to a time range of 138 min to 140 min and of 135 to 140 min, respectively. Material 5 exhibited the lowest insulating fire temperature range of 62 to 140 min at a thickness of 100 mm.
- The best results for the material thickness of 50 mm were obtained for Material 4. Moreover, at a thickness of 15 mm, Material 4 exhibited the longest time in maintaining the effectiveness of insulating against fire temperatures (from 30 to 38 min). This may be due to the fact that Material 4 exhibits the greatest density (826.94 ± 31.37 kg/m^3^) of all the tested materials.
- However, based on the results, the fire resistance of foamed concrete does not only depend on the density of the material. A change in the manufacturing process to continuous foam feeding may provide similar results in most of the thicknesses studied.

Future research should focus on obtaining a more stable structure of foam concrete, which will eliminate the possible influence of inhomogeneities on the test results. In addition, other criteria, such as the porosity of foam concrete, should be investigated in terms of influence on fire resistance.

## Figures and Tables

**Figure 14:**
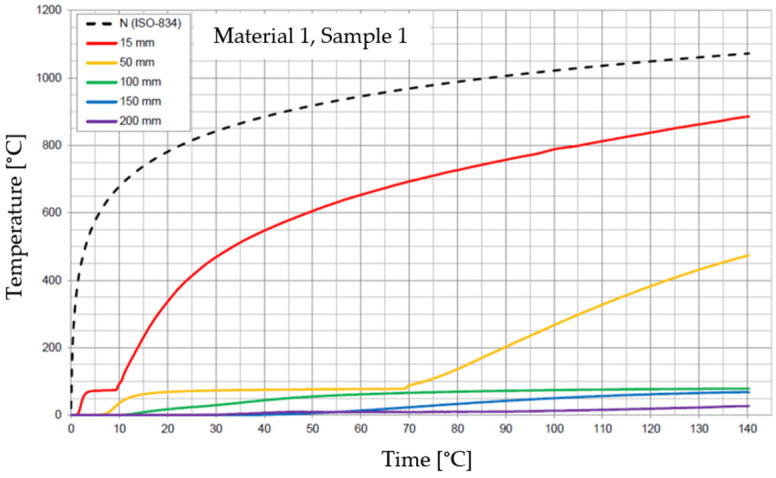
Temperature time courses for Sample 1.1.

**Figure 15:**
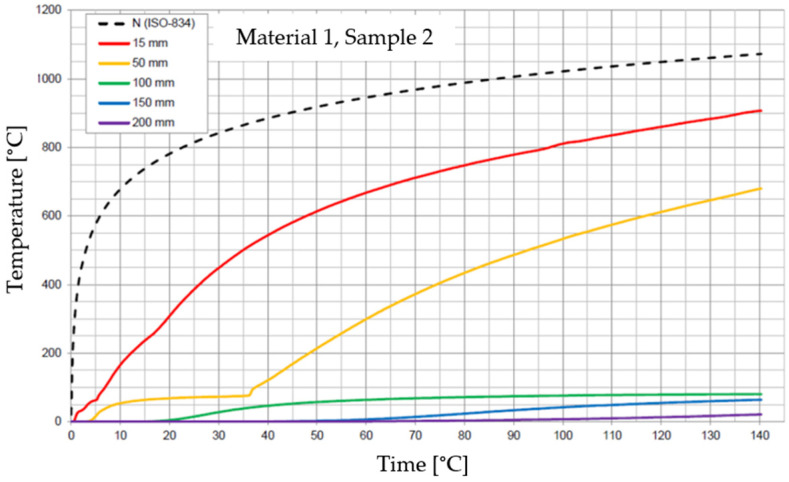
Temperature time courses for Sample 1.2.

**Figure 16:**
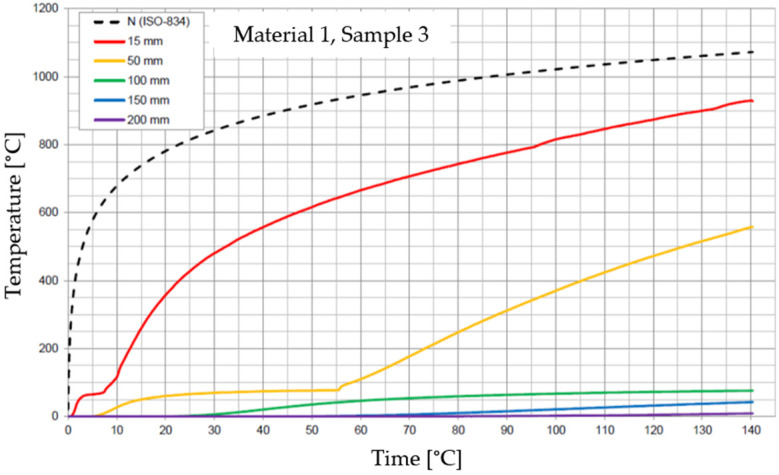
Temperature time courses for Sample 1.3.

**Table 1:** The most frequently occurring keywords based on keyword analysis.

Keyword	Links	Total Link Strength	Occurrences
Elevated temperatures	40	233	90
Beam	36	135	66
Columns	31	140	62
High-performance concrete	34	222	61
Compressive strength	33	200	59
Polypropylene fibers	36	220	56
Fire	31	101	53
Steel	35	125	50
Microstructure	36	187	48
Fire resistance	29	110	47

**Table 2:** Comparison of foam concrete and other types of concrete.

Concrete Type	Foam Concrete	Autoclaved Cellular Concrete	Hollow Concrete Blocks	Plain Concrete
Composition	Water, sand, cement, and foaming agents + aggregates [13]	Lime, cement, sand, aluminum powder + energy stimulus [14]	Sand, water, cement + mold [15]	Sand, water, cement + mold [16]
Manufacturing method; equipment	Prefabrication or in situ; mortar mixer and foam generator [13]	Prefabrication; high pressure autoclave [17]	Prefabrication [15]	Prefabrication or in situ [16]
Compressive strength, MPa	<51.18 [18]	<12 [14]	<40.5 [19]	20–50 [20]
Density, kg/m^3^	• Grouting, thermal insulation: 300–600 • Non-load bearing structures: 600–1200 • Load-bearing structures: 1200–1600 [13]	Non-structural elements: 700 [14]	Structural and non-structural elements: 1700–2000 [21]	Structural elements: 2400 [22]
Shape and size of element	Any shape [23]	Any shape in a prepared mold [17]	Different sizes in block forms [15]	Any shape [16]
Concrete compaction	Not required [13]	Not required [24]	Required [15]	Required [16]
Thermal conductivity, W/mK	0.1–0.7 [25]	0.07–0.17 [24]	<1.75 [26]	1.4–3.6 [20]
Acoustic properties	Enhanced [13]	Enhanced [14]	Good [27]	Good [27]
Ease of manufacturing and processing	Enhanced; ease of drilling and cutting [23]	Enhanced [17]	Normal [15]	Normal [16]
Environmental impact	Pollution-free; lower energy consumption [23]	High energy consumption [14]	Pollution-free [15]	Pollution-free if well cured [28]

**Table 3:** Composition of foam concrete materials.

Element	Material 1	Material 2	Material 3	Material 4	Material 5
Water (L)	7.5	7.5	6	7.5	-
Cement 42.5 R (kg)	14	14	-	14	-
Cement 32.5 R (kg)	-	-	13.5	-	-
Cement grout (m^3^)	-	-	-	-	2
Foam (0.4 MPa/3% conc.)	30	24	24	12	-
Foam (g/L) ^1^	-	-	-	-	80
Ash (kg)	-	-	-	-	80

^1^ continuous foam feeding.

**Table 4:** Weight and density of samples.

Material	Sample	Weight, kg	Density, kg/m^3^	Average Density, kg/m^3^
1	1	15.13	617.55	624.08 ± 11.31
	2	15.13	617.55	
	3	15.61	637.14	
2	1	16.38	668.57	659.86 ± 8.57
	2	15.96	651.43	
	3	16.16	659.59	
3	1	11.39	464.90	490.75 ± 22.69
	2	12.43	507.35	
	3	12.25	500.00	
4	1	19.38	791.02	826.94 ± 31.37
	2	20.60	840.82	
	3	20.80	848.98	
5	1	7.16	292.24	302.04 ± 8.53
	2	7.54	307.76	
	3	7.50	306.12	

**Table 5:** Summarized results of the foam concrete fire resistance tests.

Thickness, mm		15	50	100	150	200
Material	Average Density, kg/m^3^	Effectiveness of Insulating Fire Temperatures, min				
1	624.08 ± 11.31	11–13	47–87	140	140	140
2	659.86 ± 8.57	9–24	38–130	138–140	140	140
3	490.75 ± 22.69	12–14	53–63	135–140	140	140
4	826.94 ± 31.37	30–38	140	140	140	140
5	302.04 ± 8.53	11–13	60–70	62–140	140	140

## Data Availability

The raw data supporting the conclusions of this article will be made available by the authors on request.
